# Data on proteome of *Mycoplasma hominis* cultivated with arginine or thymidine as a carbon source

**DOI:** 10.1016/j.dib.2020.106034

**Published:** 2020-07-17

**Authors:** Tatiana A. Semashko, Daria V. Evsyutina, Valentina G. Ladygina, Aleksandr I. Zubov, Irina V. Rakovskaya, Sergey I. Kovalchuk, Rustam H. Ziganshin, Olga V. Pobeguts

**Affiliations:** a Federal Research and Clinical Center of Physical-Chemical Medicine of Federal Medical Biological Agency, Moscow, Russia; b Gamaleya National Research Center of Epidemiology and Microbiology, Moscow, Russia; c Shemyakin-Ovchinnikov Institute of Bioorganic Chemistry, Moscow, Russia

**Keywords:** Mycoplasma hominis, Proteome, Cultivation conditions, Carbon source, Arginine, Thymidine

## Abstract

*Mycoplasma hominis* is an opportunistic bacterium that can cause acute and chronic infections of the urogenital tract. This bacterium, like all other Mycoplasma species, is characterized by the reduced genome size, and, consequently, reduction of the main metabolic pathways. *M. hominis* cells cannot effectively use glucose as a carbon and energy source. Therefore, the main pathway of energy metabolism is the arginine dihydrolase pathway. However, several bacteria can use nucleosides as the sole energy source. Biochemical studies using Salmonella typhimurium have shown that three enzymes (thymidine phosphorylase, phosphopentose mutase and deoxyribose-phosphate aldolase) are involved in the thymidine catabolic pathway. All these enzymes are present in *M. hominis*. For understanding changes in the energy metabolism of *M. hominis* we performed shotgun proteome analysis of *M. hominis* cells in liquid medium with arginine or thymidine as a carbon source. LC-MS analysis was performed with an Ultimate 3000 Nano LC System (Thermo Fisher Scientific) coupled to a Q Exactive HF benchtop Orbitrap mass spectrometer (Thermo Fisher Scientific) via a nanoelectrospray source (Thermo Fisher Scientific). Data are available via ProteomeXchange with identifier PXD018714 (https://www.ebi.ac.uk/pride/archive/projects/PXD018714).

Specifications table

**Table:** 

Subject	Biology
Specific subject area	Proteomics
Type of data	LC-MS/MS data and identification data
How data were acquired	LC-MS analysis is performed with an Ultimate 3000 Nano LC System (Thermo Fisher Scientific) coupled to a Q Exactive HF benchtop Orbitrap mass spectrometer (Thermo Fisher Scientific) via a nanoelectrospray source (Thermo Fisher Scientific).
Data format	Raw and analyzed data
Parameters for data collection	Shotgun proteomes for *M. hominis* cells growing in two conditions of culturing.
Description of data collection	*M. hominis* cells growing in culture with arginine or thymidine as carbon source at log phase were collected, and their total proteomes were analyzed by shotgun proteomics in three biological replicates.
Data source location	Research and Clinical Center of Physical-Chemical Medicine, Moscow, Russian Federation
Data accessibility	Data were deposited to the PRIDE repository: Project accession: PXD018714 Project https://www.ebi.ac.uk/pride/archive/projects/PXD018714

## Value of the data

• This dataset provides proteome data for *M. hominis* cells growing in culture with arginine or thymidine as carbon source.
• These data can be interesting for the investigation of interaction with the environment of opportunistic bacteria *M. hominis*.
• These data can be interesting for the investigation of metabolism of *M. hominis* and another mycoplasma species that can be a model of a minimal cell.

## 1 Data description

*Mycoplasma hominis* is a human opportunistic bacterium that can cause acute and chronic infections of the urogenital tract [1]. Like all other Mycoplasma species, it is characterized by the reduced genome size (about 550 ORFs), and, consequently, reduction of the main metabolic pathways. *M. hominis* cells cannot effectively use glucose as a carbon and energy source. Therefore, the main pathway of energy metabolism is the arginine dihydrolase pathway, which includes arginine deiminase, ornithine carbamoyltransferase and carbamate kinase [2]. However, *M. hominis* cells can utilize nucleosides. In thymidine catabolic pathway the thymidine phosphorylase, phosphopentose mutase and deoxyribose-phosphate aldolase are involved [3].

We performed shotgun proteome analysis of *M. hominis* cells in liquid medium with arginine or thymidine as a carbon and energy source. LC-MS analysis was performed with an Ultimate 3000 Nano LC System (Thermo Fisher Scientific) coupled to a Q Exactive HF benchtop Orbitrap mass spectrometer (Thermo Fisher Scientific) via a nanoelectrospray source (Thermo Fisher Scientific). Protein identification and label-free quantification were made by PEAKS software. The data have been deposited to the ProteomeXchange Consortium via the PRIDE partner repository with the identifier PXD018714.

**Fig. 1:**
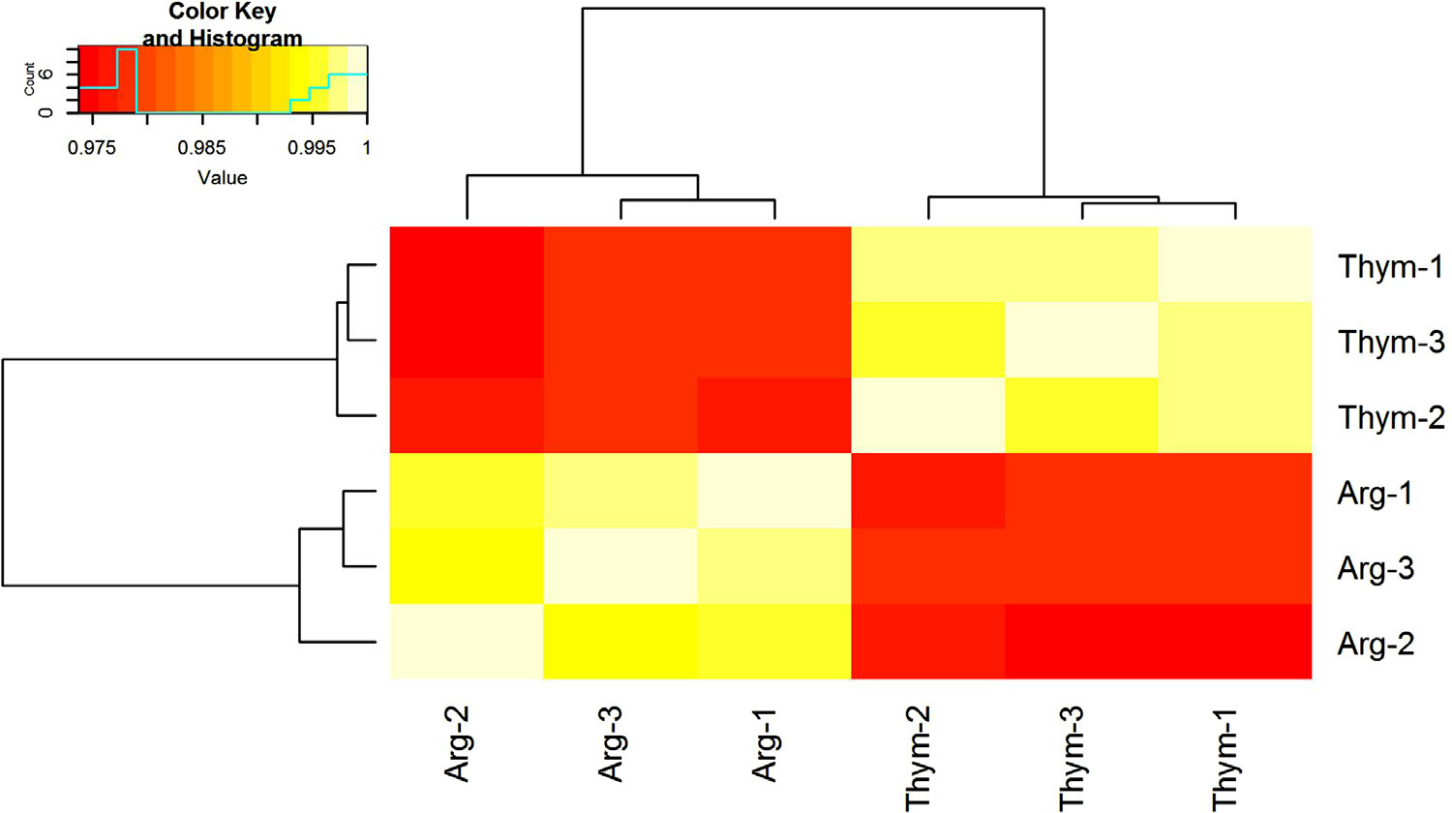
Similarity of proteomic data between samples.

**Fig. 2:**
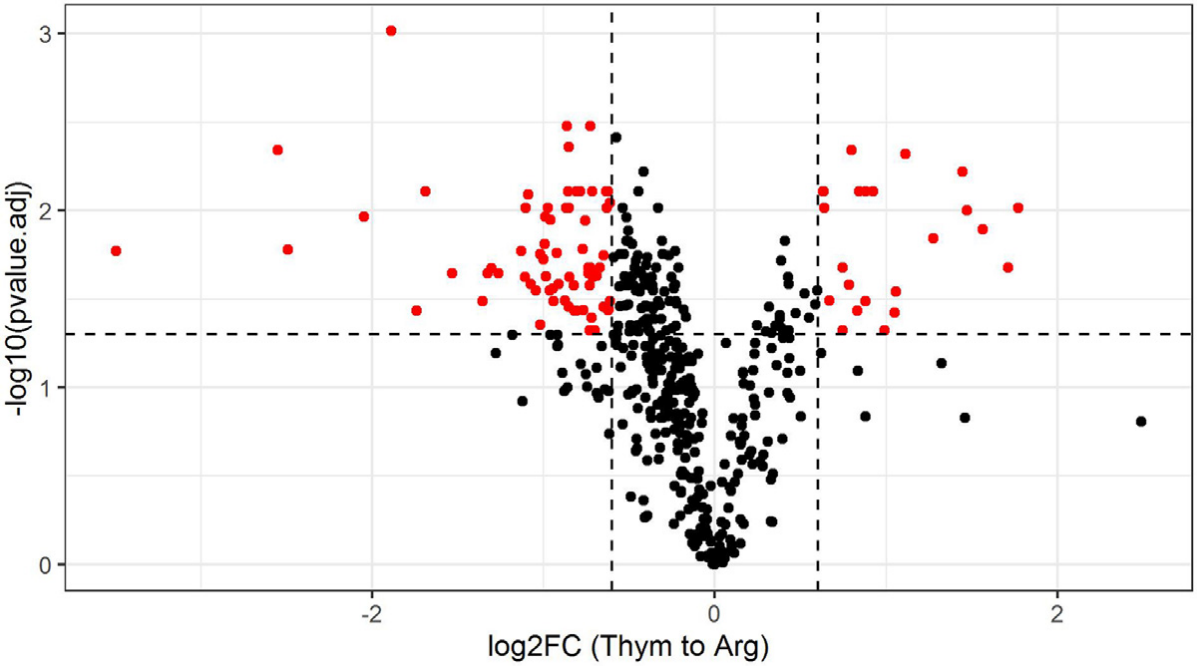
Different protein abundance for M. hominis growing with thymidine to growing with arginine.

Totally, 466 proteins were identified in both cases of *M. hominis* culturing with arginine or thymidine as carbon source (table S1). Obtained datasets show good reproducibility between biological replicas as demonstrated by the heatmap (Fig. 1). Range of differences in the protein changes is shown on volcano plot (Fig. 2). Proteins, significantly changed between aforementioned conditions (fold change > 1.5, *t*-test with Benjamini–Hochberg correction, *p* < 0.05), are presented in Table 1. All the above-mentioned enzymes from the metabolic pathways of arginine and thymidine utilization have been identified. When growing on thymidine, the only thymidine phosphorylase abundance was increased by 1.8 times, the abundance of other enzymes did not significantly change.

**Table 1:** Significantly changed proteins for *M. hominis* growing in different growth conditions. Log2FC – logarithm of fold change ratio for growth with thymidine to growth with arginine.

Accession	Log2FC	P value adj.	Description
WP_012855601.1	1.77	0.010	hypothetical protein
WP_012855692.1	1.71	0.021	pyruvate kinase
WP_012855537.1	1.56	0.013	hypothetical protein
WP_012855507.1	1.47	0.010	transcriptional regulator MraZ
WP_012855710.1	1.44	0.006	ABC transporter ATP-binding protein
WP_012855796.1	1.27	0.014	30S ribosome-binding factor RbfA
WP_012855529.1	1.11	0.005	N(G) N(G)-dimethylarginine dimethylaminohydrolase
WP_012855730.1	1.06	0.029	tRNA uridine-5-carboxymethylaminomethyl(34) synthesis GTPase MnmE
WP_012855297.1	1.05	0.038	chaperone protein ClpB
WP_012855435.1	0.99	0.048	hypothetical protein
WP_012855457.1	0.92	0.008	NAD(+) synthase
WP_012855371.1	0.88	0.033	hypothetical protein
WP_012855709.1	0.88	0.008	hypothetical protein
WP_012855608.1	0.84	0.008	thymidine phosphorylase
WP_012855370.1	0.83	0.037	NUDIX domain-containing protein
WP_012855640.1	0.80	0.005	DUF885 domain-containing protein
WP_012855422.1	0.78	0.026	30S ribosomal protein S12
WP_012855390.1	0.74	0.048	single-stranded DNA-binding protein
WP_012855783.1	0.74	0.021	type I glyceraldehyde-3-phosphate dehydrogenase
WP_012855759.1	0.67	0.032	serine/threonine-protein phosphatase
WP_012855649.1	0.64	0.010	protein LemA
WP_012855632.1	0.64	0.008	lactate dehydrogenase
WP_012855504.1	0.63	0.008	cell division protein FtsZ
WP_012855582.1	−0.61	0.033	leucine–tRNA ligase
WP_012855330.1	−0.61	0.009	Lmp1 protein
WP_012855717.1	−0.62	0.036	GTPase Era
WP_012855408.1	−0.62	0.008	alanine–tRNA ligase
WP_012855532.1	−0.63	0.037	XRE family transcriptional regulator
WP_012855641.1	−0.63	0.010	oligoendopeptidase F
WP_012855592.1	−0.64	0.008	hypothetical protein
WP_012855418.1	−0.65	0.018	RluA family pseudouridine synthase
WP_012855433.1	−0.65	0.035	rRNA pseudouridine synthase
WP_012855356.1	−0.67	0.021	hypothetical protein
WP_012855477.1	−0.69	0.023	NAD-dependent DNA ligase LigA
WP_012855316.1	−0.70	0.022	1-acyl-sn-glycerol-3-phosphate acyltransferase
WP_012855626.1	−0.70	0.022	ABC transporter ATP-binding protein
WP_012855757.1	−0.70	0.048	16S rRNA (guanine(966)-N(2))-methyltransferase RsmD
WP_012855399.1	−0.71	0.024	TlyA family rRNA (cytidine-2′-O)-methyltransferase
WP_012855628.1	−0.72	0.008	16S rRNA (guanine(527)-N(7))-methyltransferase RsmG
WP_012855539.1	−0.72	0.040	DegV family EDD domain-containing protein
WP_012855668.1	−0.73	0.021	YihA family ribosome biogenesis GTP-binding protein
WP_012855402.1	−0.73	0.003	DNA polymerase IV
WP_012855542.1	−0.73	0.048	DNA-directed RNA polymerase subunit beta'
WP_012855470.1	−0.73	0.026	deoxyguanosine kinase
WP_012855471.1	−0.73	0.021	hypoxanthine phosphoribosyltransferase
WP_020002555.1	−0.74	0.021	DUF402 domain-containing protein
WP_012855437.1	−0.74	0.023	peptide chain release factor 1
WP_041359585.1	−0.76	0.011	hypothetical protein
WP_012855413.1	−0.77	0.037	tRNA1(Val) (adenine(37)-N6)-methyltransferase
WP_012855453.1	−0.77	0.016	alcohol dehydrogenase
WP_012855553.1	−0.78	0.008	type I methionyl aminopeptidase
WP_012855662.1	−0.81	0.008	RpiB/LacA/LacB family sugar-phosphate isomerase
WP_012855625.1	−0.81	0.037	hypothetical protein
WP_012855642.1	−0.82	0.037	adenine phosphoribosyltransferase
WP_080569061.1	−0.82	0.026	hypothetical protein
WP_012855743.1	−0.85	0.024	ribonuclease III
WP_012855497.1	−0.85	0.004	ribosome biogenesis GTPase YlqF
WP_012855518.1	−0.85	0.035	hypothetical protein
WP_012855403.1	−0.85	0.010	nicotinate (nicotinamide) nucleotide adenylyltransferase
WP_012855376.1	−0.86	0.008	DNA polymerase III subunit
WP_012855694.1	−0.86	0.003	exodeoxyribonuclease V subunit alpha
WP_012855699.1	−0.87	0.010	tRNA (guanosine(46)-N7)-methyltransferase TrmB
WP_012855514.1	−0.88	0.032	RNA methyltransferase
WP_012855721.1	−0.91	0.026	spermidine/putrescine ABC transporter permease
WP_012855655.1	−0.92	0.017	2 3-bisphosphoglycerate-independent phosphoglycerate mutase
WP_012855740.1	−0.94	0.033	hypothetical protein
WP_012855290.1	−0.94	0.028	TatD family deoxyribonuclease
WP_012855419.1	−0.96	0.011	hypothetical protein
WP_012855591.1	−0.96	0.028	hypothetical protein
WP_012855323.1	−0.98	0.010	hypothetical protein
WP_012855798.1	−0.99	0.023	23S rRNA (pseudouridine(1915)-N(3))-methyltransferase RlmH
WP_012855725.1	−0.99	0.011	RDD family protein
WP_080569060.1	−0.99	0.015	hypothetical protein
WP_012855552.1	−1.00	0.019	translation initiation factor IF-1
WP_012855289.1	−1.02	0.018	ribosomal RNA small subunit methyltransferase A
WP_012855634.1	−1.02	0.044	hypothetical protein
WP_012855765.1	−1.05	0.028	hypothetical protein
WP_012855334.1	−1.08	0.026	hypothetical protein
WP_012855502.1	−1.09	0.008	16S rRNA (uracil(1498)-N(3))-methyltransferase
WP_012855595.1	−1.11	0.010	putative immunoglobulin-blocking virulence protein
WP_041359577.1	−1.11	0.024	hypothetical protein
WP_012855512.1	−1.13	0.017	tRNA pseudouridine(55) synthase TruB
WP_012855378.1	−1.26	0.023	30S ribosomal protein S20
WP_012855580.1	−1.30	0.021	signal peptidase II
WP_012855576.1	−1.33	0.023	hypothetical protein
WP_012855409.1	−1.36	0.033	Holliday junction resolvase RuvX
WP_012855438.1	−1.53	0.023	peptide chain release factor N(5)-glutamine methyltransferase
WP_012855523.1	−1.69	0.008	hypothetical protein
WP_012855355.1	−1.74	0.037	hypothetical protein
WP_012855495.1	−1.89	0.001	signal recognition particle protein
WP_012855794.1	−2.05	0.011	DUF448 domain-containing protein
WP_012855696.1	−2.49	0.017	tRNA lysidine(34) synthetase TilS
WP_012855645.1	−2.55	0.005	hypothetical protein
WP_012855350.1	−3.49	0.017	hypothetical protein

## 2 Experimental design, materials, and methods

### 2.1 Cell cultivation

*M. hominis* H34 strain was grown on Brain Heart Infusion (DIFCO, USA) supplemented with 10% horse serum (Biolot, Russia), 1% yeast extract (Helicon, Russia), penicillin (Sintez, Russia) with a final concentration 500 units/ml with the addition of 1% arginine or thymidine as a carbon source. The culture was grown at 37 °C till log-phase for 48 h with arginine or 96 h with thymidine carbon source.

### 2.2 Protein extraction

Aliquots (10 ml) of log-phase growing cells of *M. hominis H34* were collected by centrifugation at 12,000 g at 4 °C for 10 min. Then cells were washed twice by addition of 1 ml cold PBS buffer and centrifugation at 12,000 g at 4 °C for 10 min, 10 μl of 10% sodium deoxycholate (DCNa) and 0.5 μl nuclease mix (GE Healthcare, USA) was added to the cell pellet. After incubation for 1 hour at 4 °C, the sample was resuspended in 100 µl 100 mM Tris-HCl buffer (pH 8.0) containing 0.1% DCNa, 8 M urea and 2.5 mM EDTA. After incubation for 20 min the sample was centrifuged at 16,000 g for 10 min at 4 °C to remove intact cells and debris. The supernatant was collected, and protein concentration was measured using BCA Assay Kit (Sigma-Aldrich, USA).

### 2.3 Protein preparation to shotgun proteomic

Disulfide bonds were reduced in supernatant (containing 200 μg of total protein) by the addition of Tris(2-carboxyethyl)phosphine hydrochloride (TCEP) (Sigma-Aldrich, USA) to a final concentration of 5 mM and reaction was incubated for 60 min at 37 °C. To alkylate free cysteines, chloroacetamide (Sigma-Aldrich, USA) was added to a final concentration of 30 mM and placed at room temperature in the dark for 30 min. The step of adding TCEP was repeated. Then the sample was diluted 6-fold with 50 mM Tris-HCl, pH 8.0 with 0.01% DCNa. Trypsin Gold (Promega, USA) was added for a final trypsin:protein ratio of 1:50 (w/w) and incubated at 37 °C overnight. To stop trypsinolysis and degrade the acid-labile DCNa, trifluoroacetic acid (TFA) was added to the final concentration of 0.5% (v/v) (the pH should be less than 2.0), incubated at 37 °C for 45 min and the samples were centrifuged at 14,000 g for 10 min to remove the DCNa. Peptide extract was desalted using a Discovery DSC-18 Tube (Supelco, USA) according to the manufacturer protocol. Peptides were eluted with 1 ml of 75% acetonitrile in water containing 0.1% TFA, dried in an Acid-Resistant CentriVap Benchtop Vacuum concentrator (Labconco, USA) and resuspended in 3% acetonitrile in water containing 0.1% TFA to the final concentration of 5 μg/μl.

### 2.4 LC-MS analysis

LC-MS analysis was carried out on an Ultimate 3000 RSLC nano HPLC system connected to a QExactive Plus mass spectrometer (Thermo Fisher Scientific, USA). Samples were loaded to a home-made trap column 20×0.1 mm, packed with Inertsil ODS3 3 μm sorbent (GL Sciences, Japan), in the loading buffer (2% ACN, 98% H_2_O, 0.1% TFA) at 10 μl/min flow and separated at RT in a home-packed fused-silica column 500×0.1 mm packed with Reprosil PUR C18AQ 1.9 (Dr. Maisch, Germany) into the emitter prepared with P2000 Laser Puller (Sutter, USA) [4]. Samples were eluted with a linear gradient of 80% ACN, 19.9% H_2_O, 0.1% FA (buffer B) in 99.9% H_2_O, 0.1% FA (solvent A) from 4 to 36% of solvent B in 1 h at 0.44 μl/min flow at RT.

MS data were collected in DDA mode. MS1 parameters were as follows: 70 K resolution, 350–2000 scan range, max injection time 50 ms, AGC target 3 × 10^6^. Ions were isolated with 1.4 m/z window and 0.2 m/z offset targeting 10 highest intensity peaks of +2 to +6 charge, 8 × 10^3^ minimum AGC, preferred peptide match and isotope exclusion. Dynamic exclusion was set to 40 s. MS2 fragmentation was carried out in HCD mode at 17,5 K resolution with 27% NCE. Ions were accumulated for max 45 ms with target AGC 1 × 10^5^.

### 2.5 Protein identification and quantitative analysis

Identification and label-free quantification analysis were performed with PEAKS software [5] with default settings. The data were searched against *M. hominis* ATCC 23,114 NCBI database and have been deposited to the ProteomeXchange Consortium via the PRIDE [6] partner repository with the dataset identifier PXD018714 and project 10.6019/PXD018714 (http://dx.doi.org/10.6019/PXD018714, https://www.ebi.ac.uk/pride/archive/projects/PXD018714). Further calculations and visualizations were made in R [7].

## Declaration of Competing Interest

The authors declare that they have no known competing financial interests or personal relationships which have, or could be perceived to have, influenced the work reported in this article.

## Appendix Supplementary materials

Image, application 1

Image, application 2
